# Osborn Waves: History and Significance

**Published:** 2004-01-01

**Authors:** Mitsunori Maruyama, Yoshinori Kobayashi, Eitaroh Kodani, Yoshiyuki Hirayama, Hirotsugu Atarashi, Takao Katoh, Teruo Takano

**Affiliations:** First Department of Internal Medicine, Nippon Medical School, Tokyo, Japan

**Keywords:** Osborn wave, J wave, hypothermia, hypercalcemia, myocardial ischemia, ventricular fibrillation, history, clinical significance

## Introduction

**Figure 1:**
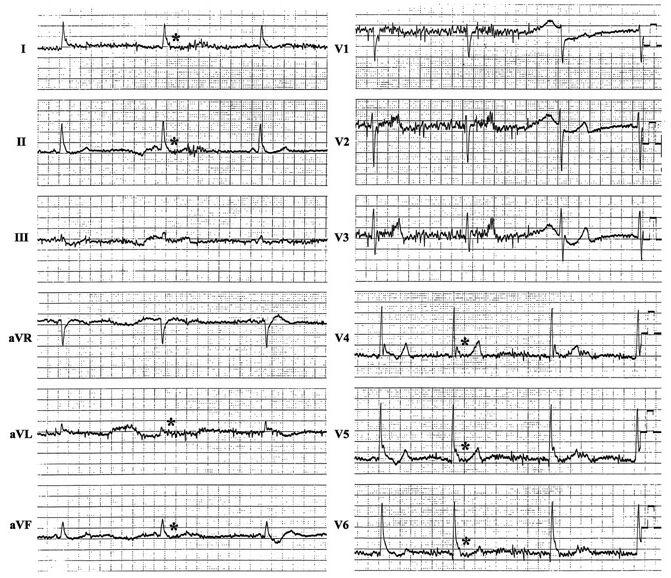
Twelve-lead ECG obtained in a 56-year-old man with a core body temperature of 32.7º C because of accidental exposure to cold. The tracing shows sinus bradycardia, prolonged QT intervals, a base-line artifact due to muscle tremors and distinctive and characteristic Osborn waves (asterisks).

The Osborn wave is a deflection with a dome or hump configuration occurring at the R-ST junction (J point) on the ECG (Figure 1). In the historical view, different names have been used for this wave in the medical literature, such as “camel-hump sign”, “late delta wave”, “hathook junction”, “hypothermic wave”, “J point wave”, “K wave”, “H wave” and “current of injury” [1].  Although there is no definite consensus about terminology of this wave, either “Osborn wave” or “J wave” are the most commonly used names for this wave in the current clinical and experimental cardiology.  The Osborn wave can be generally observed in hypothermic patients [1-4], however, other conditions have been reported to cause Osborn waves, such as hypercalcemia [5], brain injury [6], subarachnoid hemorrhage [7], cardiopulmonary arrest from oversedation [8], vasospastic angina [9], or idiopathic ventricular fibrillation [10-12]. Our knowledge about the link between the Osborn waves and cardiac arrhythmias remains sparse and the arrhythmogenic potential of the Osborn waves is not fully understood. In this paper, we present a historic review of Osborn waves and discuss their clinical significance in the various clinical settings.

## Historic Overview of the Osborn Waves

In 1953, Osborn [13] studied the effect of hypothermia on the respiratory and cardiac function in dogs. Experimentally-induced hypothermia caused the development of a distinct deflection at the J point on the ECG, which he called “current of injury”. Earlier than the Osborn’s description, similar deflections on the ECG had already been described in 1920 and 1922 by Kraus [14,15] in hypercalcemic conditions, and in 1938 by Tomashewski [16] in a hypothermic patient. Although there had been several reports regarding an alternation in the ECG at the J point prior to Osborn’s article, this deflection came to be called the “Osborn wave” in honor of his systematic and excellent work. Osborn considered acidemia induced by hypothermia as a primary cause of the Osborn wave, because it disappeared if the arterial pH was normalized by hyperventilation during the same degree of cooling [13]. In 1959, Emslie-Smith et al [17]. found differences in the endocardial and epicardial responses of the ventricular myocardium to cold, and the Osborn wave was more prominent in the epicardial than endocardial leads. Also, they questioned the participation of acidosis in the genesis of the Osborn wave on the basis of their observation that the Osborn waves appeared in hypothermic dogs irrespective of the blood pH. In the same year, West et al [18]. confirmed that the spike and dome pattern could be recorded by a microelectrode technique in the canine epicardial action potential. The resulting notch in the action potential was rate sensitive and markedly accentuated under hypothermic conditions. Earlier studies attributed the Osborn waves to a variety of factors, including anoxia, injury current, acidosis, delayed ventricular depolarization and early ventricular repolarization [1,13,17,19,20].

In 1988, Litovsky and Antzelevitch [21] proposed a difference in the electrophysiology of the ventricular epicardium and endocardium as the basis for the Osborn waves. The 4-aminopyridine sensitive transient outward current (I_to_) was shown to be prominent in canine ventricular epicardium, but not in the endocardium. The more conspicuous notched configuration of the epicardial action potential was supposed to produce a transmural voltage gradient during ventricular activation that manifested as the Osborn wave in the ECG. In 1996, Yan and Antzelevitch [22] elegantly clarified their hypothesis using an arterially perfused canine ventricular wedge model, which made it possible to simultaneously record transmembrane action potentials from several sites across the ventricular wall together with a transmural ECG. A highly significant correlation was shown between the amplitude of the epicardial notch and the amplitude of the Osborn wave recorded during several interventions, including hypothermia, premature stimulation, and block of I_to_ by 4-amionopyridine. In other studies, they demonstrated that a hypercalcemic [23] or ischemic condition [24], that had been reported to trigger the appearance of the Osborn waves [5,9],  accentuated the epicardial action potential notch. They had also reported that there was a difference in the electrophysiological response of the epicardium and endocardium to acetylcholine and isoproterenol [25], which might explain the occurrence of the Osborn waves in patients with neurological disorders [6,7].

Although they noted that the conduction time across the ventricular wall and the sequence of the ventricular activation were important determining factors in the manifestation of the Osborn waves [22], the results from their recent studies indicated that the primary cause of the Osborn waves seemed to result from the transmural voltage gradient associated with the heterogeneous expression of I_to_ in the ventricle.

## Clinical Significance of the Osborn Waves: Arrhythmogenic Considerations

**Figure 2:**
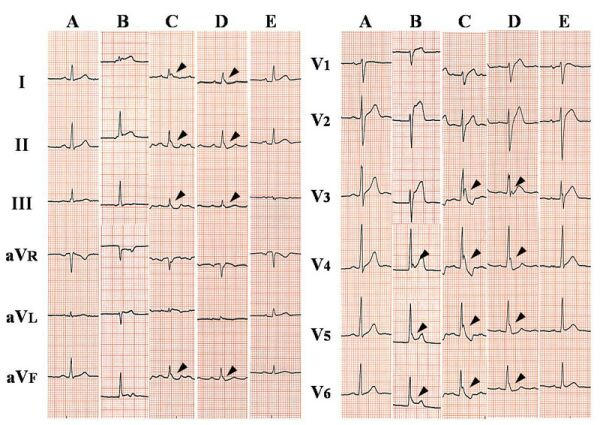
The time course of twelve-lead ECG in a 52-year-old man with vasospastic angina. ECGs were obtained prior to the ischemic attack (A), at the onset of chest pressure (B), immediately before ventricular fibrillation (C), after defibrillation and administration of intravenous lidocaine and magnesium (D), and 2 days after the episode (E). Osborn waves (arrowheads) were best seen in the inferior and lateral leads around the occurrence of ventricular fibrillation. In contrast to hypothermic patients, the tracing shows sinus tachycardia and short QT intervals.

In Osborn’s report, the presence of a deflection at the J point which he called “current of injury” heralded ventricular fibrillation and was a very bad prognostic sign in hypothermic dogs [13]. Fleming and Muir [26] confirmed the association of the Osborn waves with ventricular fibrillation in hypothermic patients. On the other hand, some reports clarified there was no correlation between the Osborn waves and ventricular fibrillation in hypothermic conditions, thus the value of the Osborn wave in hypothermic patients as a warning sign of life-threatening arrhythmias is controversial [1,17]. As a matter of fact, the Osborn waves observed in patients with hypercalcemia and neurological disorders are not usually accompanied by rhythm disturbances [5-8]. However, the Osborn waves observed in other situations have been shown to be linked to ventricular fibrillation. Aizawa et al [10]. reported a small series of patients with ventricular fibrillation of unknown origin whose ECGs showed unusual notches at the J point that was accentuated by longer preceding cycles. Although they attributed the notch to bradycardia-dependent intraventricular block, the characteristics of the notch in regard to the morphology and its rate-dependence, were consistent with the Osborn waves, which were referred later to as Osborn waves [27]. The occurrence of ventricular fibrillation seemed to be related to the augmentation of the Osborn waves in their report; a similar phenomenon was recently documented in a patient with a non-Q wave myocardial infarction due to severe coronary vasospasms [9]. The accentuation of the Osborn waves occurred immediately before the episodes of ventricular fibrillation (Figure 2).

Brugada syndrome, which has been shown to be due in part to a genetic disorder in the sodium channels, is characterized by right precordial non-ischemic R-ST segment elevation on the ECG and sudden cardiac death due to life-threatening arrhythmias such as ventricular fibrillation. Formerly, the RS-T segment elevation in the right precordial leads on the ECG had been considered a normal variant [20], and this unique ECG change has now been recognized to be associated with sudden cardiac death [28,29]. The RS-T segment elevation in Brugada syndrome could also be regarded as a prominent J wave in the right precordial leads, and the cellular mechanism of which has been explained in the same manner as the Osborn waves. The vector of the Osborn wave tends to be toward the left and posterior, as a result, the Osborn waves can usually be seen best in the inferior and lateral precordial leads (Figure 1,2), while in the right precordial leads in Brugada syndrome. Even though the substrates generating these deflections seem to be somewhat different, the Osborn waves might have some arrhythmogenic potentials similar to Brugada syndrome.

Several mechanisms for the occurrence of ventricular arrhythmias in association with the Osborn waves have been proposed. The Osborn waves provide an index of the presence of a prominent notch in the ventricular epicardium, with a more negative potential at the end of phase 1 of the action potential. As the termination of phase 1 shifts to negative, the availability of ICa is diminished, and outward currents may overwhelm the active inward currents, resulting in a loss of the action potential dome. Heterogeneous loss of the epicardial action potential dome induces a marked increase in the dispersion of repolarization and phase 2 reentry, which can be responsible for sustained ventricular arrhythmias. Accentuation of the epicardial action potential notch, which can lead to phase 2 reentry, has been demonstrated in canine epicardium exposed to hypothermia [23], increased [Ca^2+^]_o_, simulated ischemia [24], and sodium channel blockers known to augment the J wave in Brugada syndrome [30,;31]. Triggered automaticity is the other proposed mechanism for ventricular arrhythmias in patients with Osborn waves. Intracellular Ca^2+^ overload develops in several conditions which can cause Osborn waves such as hypothermia, myocardial ischemia and hypercalcemia. Early or delayed afterdepolarizations are likely to occur and form the basis for triggered activity due to the transient inward oscillatory current in Ca^2+^ overloaded cells [1,9,23]. An autonomic imbalance which could attend myocardial ischemia as well as neurological disorders may be another precipitating factor of ventricular arrhythmias.

Although the arrhythmogenic implications of the Osborn waves are not fully understood, the existence of this characteristic deflection may represent some underlying critical conditions. The risks of the Osborn waves for ventricular arrhythmias may vary with the different background of each patient and should be considered individually. Further studies are needed to determine the true significance of the Osborn waves under various conditions in which they can be observed.
